# Effects of Chocolate Deprivation on Implicit and Explicit Evaluation of Chocolate in High and Low Trait Chocolate Cravers

**DOI:** 10.3389/fpsyg.2017.01591

**Published:** 2017-09-12

**Authors:** Anna Richard, Adrian Meule, Malte Friese, Jens Blechert

**Affiliations:** ^1^Department of Psychology, University of Salzburg Salzburg, Austria; ^2^Centre for Cognitive Neuroscience, University of Salzburg Salzburg, Austria; ^3^Department of Psychology, Saarland University Saarbrücken, Germany

**Keywords:** hedonic deprivation, food craving, chocolate, dieting, implicit preferences, Single Category Implicit Association Test, Affect Misattribution Procedure

## Abstract

Diet failures are often attributed to an increase in cravings for attractive foods. However, accumulating evidence shows that food cravings actually decrease during energy-restricting weight-loss interventions. The current study aimed at elucidating possible mechanisms that may explain how and under which circumstances food cravings in- or decrease during dieting. Specifically, decreases in food cravings during weight-loss diets may be due to effects of energy restriction (homeostatic changes) and to effects of avoiding specific foods (hedonic changes). Thus, we used a selective, hedonic deprivation (i.e., restricting intake of a specific food in the absence of an energy deficit) that precludes homeostatic changes due to energy restriction. Furthermore, interindividual differences in food craving experiences might affect why some individuals are more prone to experience cravings during dieting than others. Thus, we investigated whether a selective deprivation of chocolate would in- or decrease craving and implicit preference for chocolate as a function of trait-level differences in chocolate craving. Participants with high and low trait chocolate craving (HC, LC) refrained from consuming chocolate for 2 weeks but otherwise maintained their usual food intake. Both groups underwent laboratory assessments before and after deprivation, each including explicit (i.e., state chocolate craving) and implicit measures (i.e., Single Category Implicit Association Test, SC-IAT; Affect Misattribution Procedure, AMP). Results showed that hedonic deprivation increased state chocolate craving in HCs only. HCs also showed more positive implicit attitudes toward chocolate than LCs on the SC-IAT and the AMP irrespective of deprivation. Results help to disambiguate previous studies on the effects of dieting on food cravings. Specifically, while previous studies showed that energy-restricting diets appear to decrease food cravings, the current study showed that a selective, hedonic deprivation in the absence of an energy deficit increases food cravings. However, this effect can only be observed for individuals with high trait craving levels. Thus, if attractive foods are strictly avoided through a selective deprivation, HCs are at risk to experience craving bouts in the absence of an energy deficit. As implicit preference was unaffected by chocolate deprivation, strong implicit preference for chocolate likely characterize a stable mechanism that drives consumption in HCs.

## Introduction

Today’s food-rich environments besiege us with highly palatable foods, luring us into eating out of reward-driven and hedonic motives that override satiety-related or other homeostatic (i.e., metabolic and weight-related) processes (Lowe and Butryn, 2007). Food craving, for example, is an intense desire to consume a specific food (Weingarten and Elston, 1990; Hormes and Rozin, 2010) that can occur in the absence of hunger (Pelchat and Schaefer, 2000). Typically craved foods are tasty and energy-dense, with chocolate being the most often craved food in Western societies (Rozin et al., 1991; Weingarten and Elston, 1991; Richard et al., 2017). However, because of their high energy density, the most often craved foods are usually also the ones that individuals want to avoid in their diet (Komatsu and Aoyama, 2014).

### The Relationship between Dieting and Food Craving

Dietary restriction is a common strategy in an effort to lose weight or to prevent weight gain (e.g., Kruger et al., 2004). However, dieting does not usually result in long-term caloric restriction, weight loss, or weight maintenance (Elfhag and Rössner, 2005; Lowe, 2015). In this regard, non-adherence to diets is often attributed to the experience of food cravings (Hall and Most, 2005). Similarly, dieters report more frequent and more intense food cravings than non-dieters (Massey and Hill, 2012), and more frequent food cravings are related to lower self-reported dieting success (Meule et al., 2011, 2017), suggesting that dieting may *increase* occurrences of food cravings. However, these findings are based on cross-sectional data and it cannot be concluded with confidence that dietary restriction causes increases in food cravings.

In contrast, studies that manipulated dietary restriction would allow drawing causal inferences. Such studies showed that weight-loss interventions can actually result in a *decrease* in food cravings (Batra et al., 2013; Kahathuduwa et al., 2017; Smithson and Hill, 2017). Similarly, it has been found that fasting or adhering to a low- or very-low calorie diet can reduce cravings for high-carbohydrate and high-fat foods (Martin et al., 2006; Martin et al., 2011) and experiences of hunger (Lappalainen et al., 1990). However, weight-loss interventions also produced mixed results such that a low-carbohydrate diet increased cravings for specific foods (Jakubowicz et al., 2012).

Nevertheless, findings about the relationship between dieting and food cravings are equivocal as it is not completely clear under which circumstances food cravings increase or decrease during dieting. This might be due to complex interactions of both hedonic and homeostatic processes that are brought about by dieting. For example, it is difficult to manipulate the homeostatic state (i.e., inducing an energy deficit by caloric restriction) without corresponding changes in the hedonic system (e.g., increases in food cravings), due to manifold bidirectional links between brain structures (e.g., hypothalamus, nucleus accumbens) and ingestive/digestive systems (e.g., leptin, ghrelin, insulin; Berthoud and Morrison, 2008). By contrast, it is possible to isolate hedonic effects of dieting by means of a selective deprivation from a specific food in the absence of a caloric deficit (Lowe and Butryn, 2007).

### Decomposing Hedonic and Homeostatic Effects of Dieting

Only few studies focused on the effects of a selective *hedonic deprivation*, that is deprivation of a desired food in the absence of a caloric deficit and hence unchanged homeostatic state. For instance, a recent study addressed the effects of a selective rice deprivation on cravings for rice, which is a staple but often craved food in Japan (Komatsu et al., 2015). Participants who were instructed to abstain from eating rice (but otherwise to keep a balanced diet) reported higher rice cravings than non-deprived participants. Another study compared the effects of 1-week selective chocolate and vanilla deprivation. Compared to vanilla-deprived and non-deprived participants, chocolate-deprived individuals ate the greatest amount of chocolate after the deprivation period (Polivy et al., 2005). Similarly, a 3-day protein or carbohydrate restriction increased cravings in a selective manner: protein-deprived participants craved proteins more, but not carbohydrates while carbohydrate-deprived participants showed the reverse pattern (Coelho et al., 2006). To summarize, these studies show that a selective deprivation increases food cravings and suggest that decreases in food cravings might only be observed in diets that result in a significant energy deficit.

While these studies on the effects of a selective deprivation obviously required some degree of liking of the deprived food, the role of interindividual differences in *trait food craving* has rarely been studied. Trait food craving refers to frequent and intense experiences of cravings in general, whereas state food cravings are exclusively experienced as transient states in a particular moment (Hallam et al., 2016). Thus, trait food cravers might be particularly vulnerable to the effects of a hedonic deprivation because they continuously experience cravings for the specific food being restricted. In support of this notion, regular chocolate eaters were found to desire and consume more chocolate when being chocolate-deprived compared to when non-deprived (Blechert et al., 2014b). In another study, high and low trait chocolate cravers were each split in a deprived group, refraining from eating chocolate for 2 weeks, or a non-deprived group, maintaining regular chocolate consumption (Moreno-Domínguez et al., 2012) and it was shown that the effects of a hedonic deprivation were crucially dependent on trait-level differences in chocolate craving. However, the literature reviewed above also raises questions about why high trait chocolate cravers might be more vulnerable to experience cravings in response to chocolate and to hedonic deprivation in particular.

### Trait Food Craving and Implicit Responses toward Foods

Craved foods appeal to basic reward circuits that can be expected to influence early affective responses (Frankort et al., 2013; Wolz et al., 2017). Accordingly, neuroimaging studies found that reward-related brain regions were more activated in chocolate cravers compared to non-cravers when exposed to the sight of chocolate (Asmaro and Liotti, 2014; Richard et al., 2016; Rolls and McCabe, 2007). On a behavioral level, some studies reported an implicit approach bias toward chocolate as indicated by faster responses to chocolate–approach than chocolate–avoidance trials in an implicit association task (e.g., Kemps et al., 2013). Similarly, it has been found that high compared to low trait food cravers showed an approach bias toward high-calorie foods in an approach-avoidance task (Brockmeyer et al., 2015). However, research considering both trait food craving and implicit responses toward food is surprisingly scarce.

A range of measures are available to index implicit attitudes (Nosek et al., 2011). To capture various aspects of implicit responding, we used both a Single-Category Implicit Association Test (SC-IAT; Karpinski and Steinman, 2006) and an Affect Misattribution Procedure (AMP; Payne et al., 2005) in the current study. The SC-IAT tests implicit associations of one target category (here: chocolate) with positive relative to negative valence while avoiding the difficulty of introducing associations with a second target category (e.g., healthy counterpart of chocolate). Here, participants sort stimuli into their respective categories while response latencies are measured. In one critical block, the target category and positive valence share the same response key; in the other critical block, the target category and negative valence share the same response key. Typically, response interference is stronger in one than in the other block, resulting in longer response latencies (e.g., when responses are compatible with the participants’ preference). By contrast, the AMP is a priming-based implicit measure that assesses the misattribution of affect aroused by briefly presented primes (here: chocolate vs. neutral primes). The AMP does not rely on response latencies. Instead, participants make evaluative judgments about normatively neutral target stimuli (i.e., Chinese ideographs). Chinese ideographs tend to be judged more positively after positive relative to negative or neutral primes. Therefore, the SC-IAT and the AMP are two indirect measures to assess spontaneous affective responses, but they rely on different mechanisms: response interference in case of the SC-IAT and misattribution of affect in case of the AMP (see Deutsch and Gawronski, 2009).

A rationale for using implicit measures is that they provide information that cannot be obtained with explicit measures. Although implicit measures are often positively correlated with corresponding self-reports, these associations are usually weak (Hofmann et al., 2005; Hahn and Gawronski, 2015). Similarly, while implicit and explicit measures may show converging results when predicting behavior, the predictive power of implicit and explicit measures can also diverge under certain conditions (e.g., those that foster the impact of automatic processes on behavior determination; Friese et al., 2009; Cameron et al., 2012). Thus, we used both explicit measures via self-report (e.g., state chocolate craving) and implicit measures via a SC-IAT and AMP (i.e., implicit chocolate preference) in the current study.

### The Present Study

On the background of inconsistent findings regarding the relationship between dieting and food craving, we investigated the effects of a hedonic deprivation in high and low trait chocolate cravers. Both groups were tested at baseline of regular chocolate consumption and after they had refrained from eating chocolate for 2 weeks. Based on previous studies (Moreno-Domínguez et al., 2012; Blechert et al., 2014b), we predicted that high trait chocolate cravers would report higher state chocolate craving than low trait chocolate cravers in general (*trait* main effect) and increased state chocolate craving after chocolate deprivation in particular (*trait* × *state* interaction). Predictions were similar for implicit measures: based on previous studies on implicit approach bias toward high-calorie foods in trait food cravers (Kemps et al., 2013; Brockmeyer et al., 2015), we expected a *trait* main effect such that high trait chocolate cravers would show a higher implicit chocolate preference than low trait chocolate cravers in general. As increases in implicit food preference have been found in food-deprived participants (Seibt et al., 2007), we speculated that implicit attitudes toward chocolate may be increased after chocolate deprivation in high trait chocolate cravers in particular (*trait* × *state* interaction).

## Materials and Methods

### Participants

A total of 131 participants were recruited through flyers shared on social media platforms, the campus’ bulletin boards and student mailing lists. They completed the chocolate version of the Food Cravings Questionnaire-Trait-reduced (FCQ-T-r; Meule and Hormes, 2015) online. To recruit high and low trait chocolate cravers, participants scoring in the upper and lower tertiles of the distribution were contacted via telephone and interviewed to exclusion criteria (i.e., current dieting, food allergies). Two additional inclusion criteria were predefined for high trait chocolate cravers: chocolate liking greater than 80 on a scale ranging from 0 (*not at all*) to 100 (*a lot*) and chocolate consumption more than three times a week. Out of 86 invited participants, 74 were tested in the laboratory. However, data from 14 participants were excluded from analyses because they did not attend the second laboratory assessment (*n* = 5), did not perform the laboratory tasks correctly (*n* = 4), and due to technical failures (*n* = 5). The final sample consisted of 39 high trait chocolate cravers (eight males) and 21 low trait chocolate cravers (seven males) with similar age and body mass index (BMI). Descriptive statistics for age, BMI, hunger, chocolate liking, chocolate consumption, and trait chocolate craving are displayed in **Table 1**.

**Table 1:** Sample description with means (standard deviations) of low trait chocolate cravers (*n* = 21) and high trait chocolate cravers (*n* = 39).

	Low trait chocolate cravers	High trait chocolate cravers	Test statistics
Age (years)	24.5 (5.47)	24.0 (4.67)	*t* = -0.39, *p* = 0.698, *d* = -0.10
Body mass index (kg/m^2^)	21.6 (2.54)	22.1 (2.34)	*t* = 0.74, *p* = 0.462, *d* = 0.20
Hunger (non-deprived state)	15.7 (5.02)	15.7 (7.91)	*t* = 0.01, *p* = 0.994, *d* < 0.01
Hunger (deprived state)	15.6 (6.45)	15.6 (7.80)	*t* = -0.03, *p* = 0.980, *d* < 0.01
Chocolate liking	40.1 (22.0)	90.4 (7.58)	***t* = 9.32, *p* < 0.001, *d* = 3.28**
Usual chocolate consumption (times per week)	1.13 (0.96)	5.11 (1.54)	***t* = 11.0, *p* < 0.001, *d* = 3.14**
Food Cravings Questionnaire-Trait-reduced	18.9 (3.80)	55.1 (11.9)	***t* = 17.3, *p* < 0.001, *d* = 4.10**


*Significant differences are printed in boldface; Chocolate liking (“How much do you like chocolate in general?”) was assessed on a scale from 0 (*not at all*) to 100 (*a lot*).*

### Measures

#### Chocolate Versions of the Food Cravings Questionnaires

The chocolate-adapted version of the FCQ-T-r (Meule and Hormes, 2015) was used to measure the frequency of chocolate craving experiences. It consists of 15 items (e.g., “When I crave chocolate, I know I won’t be able to stop eating once I start.”) and responses are scored on a 6-point scale (1 [*never/not applicable*] to 6 [*always*]). Internal consistency was α = 0.973 in the current study.

The chocolate-adapted version of the Food Cravings Questionnaire-State (FCQ-S; Meule and Hormes, 2015) was used to measure intensity of current chocolate craving. Its 15 items (e.g., “I have an intense desire to eat chocolate.”) are scored on a 5-point scale (1 [*strongly disagree*] to 5 [*strongly agree*]). Internal consistencies were α = 0.932 (deprived state) and α = 0.911 (non-deprived state) in the current study.

#### Implicit Attitudes toward Chocolate

A SC-IAT (Karpinski and Steinman, 2006; Bluemke and Friese, 2008) was used to assess implicit attitudes toward chocolate. The SC-IAT consisted of a 3-block sequence: in block 1, participants practiced the categorization of positive and negative target words (20 trials), followed by two critical testing blocks (70 trials each). In the testing blocks, participants sorted stimuli into one of three categories labeled *unpleasant, pleasant*, and *chocolate*. The evaluative categories were represented by 10 negative and 10 positive words, respectively. The target category was represented by 10 chocolate pictures (retrieved from food.pics database; Blechert et al., 2014a)^1^. In every trial, a stimulus appeared and remained on the screen until the participant responded or a maximum of 1700 ms had elapsed (in which case participants were prompted to respond faster). A 150 ms inter-trial interval separated trials. Erroneous responses were signaled by a red cross. In the first testing block, *d* was the response key for negative words and *l* was the response key for positive words and chocolate pictures. In the second testing block, the assignment of chocolate pictures was reversed such that negative words and chocolate pictures shared the *d* key and positive words were sorted on the *l* key.

As both pictures and half of the words were sorted to the same side, response bias to that side might arise. Thus, the frequency of words and chocolate pictures was adjusted so that the proportion of the *d* and *l* response keys was 3:4 in the first testing block and 4:3 in the second testing block, respectively (Friese et al., 2008). Block order was the same across participants because the focus was on relative differences between high and low trait chocolate cravers and not on absolute SC-IAT effects (Gawronski, 2002).

Single-Category Implicit Association Test scores were calculated using the D600 algorithm (Greenwald et al., 2003), which calculates the mean reaction time difference between the two critical testing blocks divided by the standard deviation of all correct response times within both blocks and penalizes errors with a 600 ms addition. Higher SC-IAT scores indicate a more positive implicit attitude toward chocolate. Internal consistency was calculated by creating four mutually exclusive subsets of trials and SC-IAT scores were then calculated separately for all subsets. Across subsets, internal consistencies were α = 0.823 (deprived state) and α = 0.755 (non-deprived state) in the current study. Based on individual- and block-specific *z*-scores, trials with values ± 3.29 were excluded (0.80% in block 2, 1.20% in block 3). Non-responses and responses < 400 ms (3.30%) were eliminated (Greenwald et al., 2003).

The AMP (Payne et al., 2005) served as a second indicator of implicit attitudes toward chocolate. The stimulus set contained 60 Chinese ideographs (retrieved from Payne et al., 2005), 15 chocolate pictures, and 15 neutral objects. Chocolate pictures and neutral objects (retrieved from food.pics database; Blechert et al., 2014a) were matched in shape and color^2^. In the practice block (6 trials), participants rated Chinese ideographs that were preceded by geometric figures. In the testing block, a prime stimulus (100 ms) was followed by an interstimulus interval (ISI; blank screen) with either 100 ms (30 trials) or 1500 ms duration (30 trials). Then, a Chinese ideograph was presented for 200 ms followed by a masking screen, which terminated with participants’ keyboard rating on a scale from -2 (*very unpleasant*) to 2 (*very pleasant;* response keys *x, c, n, m*). The 15 chocolate pictures and 15 neutral objects served as primes and preceded the 60 Chinese ideographs. Prime stimuli and ISI were varied according to a predetermined, random order that was identical for all participants. In line with the standard AMP (Payne et al., 2005), the difference in evaluation between chocolate and neutral prime trials served as an indicator of implicit attitudes toward these stimuli such that higher values indicate more positive implicit attitudes toward chocolate compared to neutral primes^3^. Internal consistencies were α = 0.833 (deprived state) and α = 0.848 (non-deprived state) for chocolate prime trials and α = 0.777 (deprived state) and α = 0.805 (non-deprived state) for neutral prime trials in the current study. For data analyses, responses < 300 ms (2.50%) and >3000 ms (1.90%) were excluded. For a better understanding, we recoded AMP responses to a scale from 1 to 4, with higher scores indicating more positive responses toward the Chinese ideographs.

Both implicit measures were programmed using Eprime 2.0 Professional (Psychology Software Tools, Inc., Sharpsburg, PA, United States). During the tasks, participants were seated at a distance of 50 cm to a 23-inch LCD monitor. Positive and negative words were presented in Arial Black font. Target stimuli (i.e., chocolate pictures and neutral objects) were presented with a resolution of 600 pixels × 450 pixels and control stimuli (i.e., Chinese ideographs, positive, and negative words) with a resolution of 288 pixels × 77 pixels.

#### Hunger

Current hunger was assessed with the Hunger Scale (Coletta et al., 2009). Participants indicated on a 9-point scale how hungry they were at the experiment, how strong their desire was to eat, how much food they likely could eat, and how full their stomach felt at the moment. Internal consistencies were α = 0.879 (deprived state) and α = 0.855 (non-deprived state) in the current study.

#### Palatability and Desire to Eat Chocolate

Participants were presented with the 25 chocolate pictures used in the SC-IAT and the AMP and indicated their perceived palatability of the depicted chocolate on a 10-point scale ranging from 1 (*not palatable at all*) to 10 (*very palatable*). They also rated their current desire to eat the depicted chocolate on a 10-point scale ranging from 1 (*don’t like to eat this now*) to 10 (*would like to eat this now*). Internal consistencies were α = 0.964 (deprived state) and α = 0.960 (non-deprived state) for palatability and α = 0.971 (deprived state) and α = 0.967 (non-deprived state) for desire to eat.

### Procedure

The study was approved by the ethics committee of the University of Salzburg and participants signed informed consent before commencing the study. High and low trait chocolate cravers were instructed to maintain chocolate consumption for 1 week (i.e., non-deprived phase) and to refrain from eating chocolate for 2 weeks (i.e., deprived phase; order of phases was counterbalanced across participants). To assess the success of the chocolate deprivation, high trait chocolate cravers were prompted to respond to a daily text message if they consumed chocolate on that day or not.

Participants attended laboratory assessments after the non-deprived phase and after the deprived phase. At the first laboratory assessment, participants completed questionnaires regarding state chocolate craving and hunger. After completion of the SC-IAT and the AMP, they rated the chocolate images regarding their perceived palatability and desire to eat. At the second laboratory assessment, participants completed the same state-level questionnaires and measurements. Participants were debriefed and reimbursed (course credits or participants completed the same state-level questionnaires and measurements. €30) at the end of the study.

### Statistical Analyses

Independent samples *t*-tests were conducted to investigate whether high and low trait chocolate cravers differed in age, BMI, chocolate craving, liking, and consumption. Analyses of variance for repeated measures with *trait* (high vs. low trait chocolate craving) as between-subject factor and *state* (deprived vs. non-deprived) as within-subject factor were calculated to examine deprivation effects on hunger, state chocolate craving, SC-IAT scores, palatability, and desire to eat. For the AMP, an analysis of variance for repeated measures with *trait* (high vs. low trait chocolate craving) as between-subject factor and *state* (deprived vs. non-deprived) and *stimulus category* (chocolate vs. neutral prime) as within-subject factors was calculated. Pearson product-moment correlations were computed between chocolate-related measures (i.e., state chocolate craving, SC-IAT, AMP, palatability, and desire to eat). For this, scores of these state-level measures were averaged over the deprived and non-deprived condition.

## Results

### Manipulation Checks

High and low trait chocolate cravers did not differ on hunger ratings at either laboratory assessments [both main effects and the *trait* × *state* interaction were not significant, *F*_s(1,58)_ ≤ 0.01, *p*_s_ ≥ 0.915, $\eta_{\mathrm{ps}}^{2}$ < 0.001], confirming that homeostatic state was not changed by hedonic deprivation. High trait chocolate cravers reported higher chocolate liking, chocolate consumption, and trait chocolate craving than low trait chocolate cravers prior to the study (**Table 1**). Adherence to the deprivation instruction in high trait chocolate cravers was satisfactory: 69.2% (*n* = 27) were fully compliant, whereas 28.2% (*n* = 11) ate chocolate once or twice and 2.60% (*n* = 1) ate chocolate three times during the 2-week period.

### State Chocolate Craving

**FIGURE 1:**
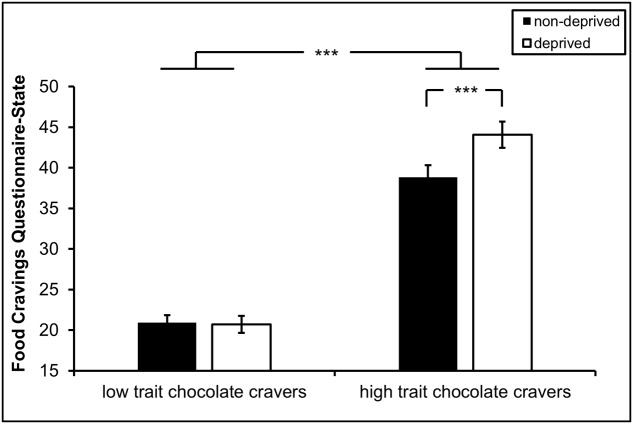
State chocolate craving displayed as a function of trait (high vs. low trait chocolate craving) and state (non-deprived vs. deprived). Error bars represent standard errors. ^∗∗∗^*p* < 0.001.

Two main effects of *trait*, *F*_(1,58)_ = 100, *p* < 0.001, $\eta_{p}^{2}$ = 0.641, and *state*, *F*_(1,58)_ = 8.48, *p* = 0.005, $\eta_{p}^{2}$ = 0.131, were modulated by a significant interaction of *trait* × *state*, *F*_(1,58)_ = 10.2, *p* = 0.002, $\eta_{p}^{2}$ = 0.154. Only high trait chocolate cravers reported higher cravings for chocolate in deprived state compared with non-deprived state, *t*_(38)_ = 4.43, *p* < 0.001, *d*_av_ = 0.73. This state effect was not seen in low trait chocolate cravers, *t*_(20)_ = -0.26, *p* = 0.797, *d*_av_ = 0.06 (**Figure 1**).

### Implicit Measures

#### Implicit Attitudes on the SC-IAT

**FIGURE 2:**
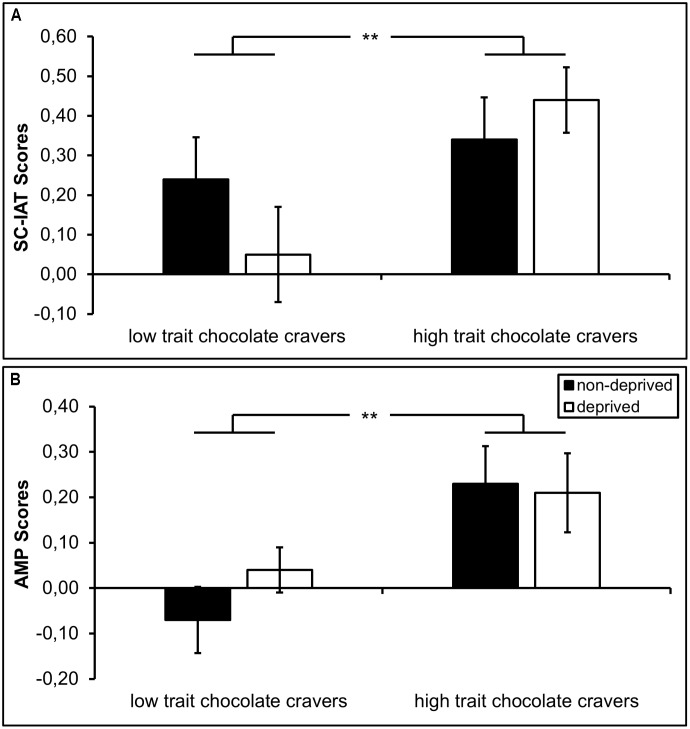
Implicit attitude toward chocolate displayed as a function of trait (high vs. low trait chocolate craving) and state (non-deprived vs. deprived). SC-IAT D600 scores were calculated and a positive score indicates a more positive implicit attitude toward chocolate **(A)**. Difference scores between chocolate and neutral prime trials in the AMP were calculated to increase analogy with the SC-IAT. A positive score indicates a more positive implicit evaluation of Chinese ideographs primed by chocolate compared to Chinese ideographs primed by neutral objects **(B)**. Error bars represent standard errors. ^∗∗^*p* < 0.01.

There was a main effect of *trait*, *F*_(1,58)_ = 7.50, *p* = 0.008, $\eta_{p}^{2}$ = 0.126, indicating that high trait chocolate cravers had more positive SC-IAT scores than low trait chocolate cravers (**Figure 2A**), indicative of a more positive implicit attitude toward chocolate. However, the main effect of *state*, *F*_(1,58)_ = 0.23, *p* = 0.637, $\eta_{p}^{2}$ = 0.004, and the *trait* × *state* interaction were not significant, *F*_(1,58)_ = 2.95, *p* = 0.092, $\eta_{p}^{2}$ = 0.054.

#### Implicit Attitudes on the AMP

There was no main effect of *trait*, *F*_(1,58)_ = 2.76, *p* = 0.103, $\eta_{p}^{2}$ = 0.050, but a main effect of *stimulus category*, *F*_(1,58)_ = 5.82, *p* = 0.019, $\eta_{p}^{2}$ = 0.101. This main effect was qualified by a significant interaction of *trait* × *stimulus category*, *F*_(1,58)_ = 7.75, *p* = 0.007, $\eta_{p}^{2}$ = 0.130. High trait chocolate cravers generally rated Chinese ideographs primed by chocolate pictures (*M* = 2.66, *SD* = 0.35) more pleasant than Chinese ideographs primed by neutral objects (*M* = 2.44, *SD* = 0.37), *t*_(38)_ = 3.89, *p* < 0.001, *d*_av_ = 0.61, whereas no differences were found in low trait chocolate cravers (chocolate: *M* = 2.43, *SD* = 0.25; neutral objects: *M* = 2.44, *SD* = 0.26), *t*_(20)_ = -0.26, *d*_av_ = -0.04 (**Figure 2B**). However, there were no main effect of and no interactions with *state* (all *F*_s_ ≤ 1.66, all *p*_s_ ≥ 0.204, all $\eta_{\mathrm{ps}}^{2}$ ≤ 0.031).

### Chocolate Ratings

#### Palatability

There was a main effect of *trait*, *F*_(1,58)_ = 80.9, *p* < 0.001, $\eta_{p}^{2}$ = 0.591, indicating higher palatability ratings for high trait chocolate cravers (*M* = 7.34, *SD* = 1.04) compared to low trait chocolate cravers (*M* = 4.35, *SD* = 1.56) across both states. The main effect of *state*, *F*_(1,58)_ = 0.89, *p* = 0.348, $\eta_{p}^{2}$ = 0.016, and the *trait* × *state* interaction were not significant, *F*_(1,58)_ = 0.11, *p* = 0.740, $\eta_{p}^{2}$ = 0.002.

#### Desire to Eat

There was a main effect of *trait*, *F*_(1,58)_ = 64.2, *p* < 0.001, $\eta_{p}^{2}$ = 0.534, indicating higher desire to eat ratings for high trait chocolate cravers (*M* = 6.52, *SD* = 1.61) compared to low trait chocolate cravers (*M* = 3.38, *SD* = 1.42) across both states. The main effect of *state*, *F*_(1,58)_ = 1.29, *p* = 0.218, $\eta_{p}^{2}$ = 0.011, and the *trait* × *state* interaction were not significant, *F*_(1,58)_ = 0.09, *p* = 0.761, $\eta_{p}^{2}$ = 0.002.

### Correlational Analyses

There were moderate to high positive correlations between chocolate picture ratings (palatability, desire to eat) and state chocolate craving. SC-IAT scores and AMP scores were each positively correlated to chocolate picture ratings and state chocolate craving (**Table 2**). Additionally, they were positively correlated with each other, supporting that both measures tap into overlapping, yet complementary, aspects of implicit attitudes toward chocolate.

**Table 2:** Descriptive statistics (means, standard deviations) of and correlations between continuous implicit and explicit study variables.

Measure	*Mean* (*SD*)	1	2	3	4	5
(1) Single Category Implicit Association Test^a^	0.31 (0.34)	–				
(2) Desire to eat ratings	5.45 (2.14)	0.439^∗∗^	–			
(3) Palatability ratings	6.32 (1.88)	0.446^∗∗∗^	0.906^∗∗∗^	–		
(4) Affect Misattribution Procedure^b^	0.14 (0.31)	0.321^∗^	0.446^∗∗^	0.416^∗∗^	–	
(5) Food Cravings Questionnaire-State	34.2 (12.5)	0.455^∗∗∗^	0.812^∗∗∗^	0.750^∗∗∗^	0.356^∗∗^	–


*^a^D600-algorithm. ^b^Difference scores of responses toward Chinese ideographs in chocolate prime trials and neutral prime trials. ^∗^*p* < 0.05, ^∗∗^*p* < 0.01, ^∗∗∗^*p* < 0.001.*

## Discussion

In the light of inconsistent findings regarding the relationship between dieting and food craving, we studied high and low trait chocolate cravers before and after 2 weeks of selective chocolate deprivation. Outcome variables included both explicit and implicit measures such as self-reported craving for chocolate and implicit preference for chocolate.

### Trait Food Craving: Underlying Systems and Theoretical Implications

As expected, state chocolate craving and chocolate ratings (i.e., desire to eat, palatability) were generally higher in high trait chocolate cravers compared with low trait chocolate cravers. This pattern of result is in line with previous studies (e.g., Rodríguez et al., 2005) and also confirms the validation of the concept trait food craving. Importantly, such main effects were not limited to self-report measures, which could be influenced by reporting biases, but extended to more implicit response systems: high trait chocolate cravers showed more positive implicit attitudes toward chocolate on both the SC-IAT and the AMP. The fact that implicit response systems are involved in trait chocolate craving is in line with neuroimaging results. For example, enhanced activity in reward-related brain regions (e.g., ventral striatum) was repeatedly found in trait chocolate cravers compared to non-cravers when exposed to the sight of chocolate stimuli (Asmaro and Liotti, 2014; Richard et al., 2016; Rolls and McCabe, 2007). This involvement of reward-related brain circuitry in trait chocolate craving may manifest behaviorally in automatic approach tendencies toward food (Kemps et al., 2013; Brockmeyer et al., 2015).

Not only that trait chocolate cravers showed higher implicit preference for chocolate in the current study, implicit measures, chocolate ratings, and state chocolate craving were positively correlated with each other as well. The strength of the correspondence between implicit and explicit measures has been shown to vary as a function of the research domain (e.g., stereotypes, anxiety) or methodological differences (e.g., stimulus properties; Hofmann et al., 2005; Nosek, 2007). Yet, in line with the current findings, it appears that more consistent relationships can be found between explicitly reported craving and implicit preference for substances of abuse and food (Roefs et al., 2011; Kemps et al., 2013).

Implicit and explicit responses represent the two core components of dual-process models (Sherman et al., 2014), whereby implicit responses are automatically activated when individuals are exposed to palatable food. Resisting attractive food requires control from conscious and reflective systems that inhibit approach behavior through activation of dieting- and health-related goals. However, successful inhibition of these temptations critically depends on situational moderators such as available self-regulatory resources or cognitive capacity (Strack and Deutsch, 2004; Hofmann et al., 2009b). In this regard, the time course of affective responses mapped in the AMP via the ISI latencies between prime and target would be of interest. Previous research found that unrestrained eaters down-regulated their immediately activated hedonic responses toward palatable food in the long (vs. short) ISI condition, whereas chronic dieters (i.e., restrained eaters) exhibited persistent positive responses in both ISI conditions (Hofmann et al., 2010). However, we could not explore these findings further because we excluded individuals who were currently dieting, thus limiting variation on restrained eating. Future research may investigate interactions between trait food craving and restrained eating to study whether positive implicit responses render dieters with high trait food craving more prone to lapses during dieting.

### Effects of Hedonic Deprivation: Implications for Dieting and Theories of Craving

As expected, state chocolate craving increased across the course of a selective chocolate deprivation only in high trait chocolate cravers. This is in line with studies that focused on hedonic deprivation (Polivy et al., 2005; Coelho et al., 2006) and trait chocolate craving in particular (Blechert et al., 2014b). However, it is in contrast to the study of Moreno-Domínguez et al. (2012), which reported increases in state chocolate craving also in deprived low trait chocolate cravers from pre- to post-test. As there were no state craving increases in low trait chocolate cravers in the current investigation, it suggests that trait food cravers are particularly prone to experience cravings during deprivation.

Deprivation effects have mostly been investigated in weight-loss interventions that included caloric deficits and accompanying weight loss (Martin et al., 2011; Batra et al., 2013). However, the psychological and physiological mechanisms that underlie the craving-reducing effects of such interventions are unclear (Kahathuduwa et al., 2017). Increases in food cravings during selective, hedonic deprivation as found in the current study and in prior studies (Coelho et al., 2006; Moreno-Domínguez et al., 2012; Blechert et al., 2014b; Komatsu et al., 2015) suggest that decreases in food cravings during energy-restricting food deprivation may be due to homeostatic changes that override hedonic processes. For example, caloric restriction may alter homeostatic gut–brain communication that results in decreased food cravings and these changes appear to be more dominant than hedonic deprivation effects that would have led to increased food cravings in the absence of an energy deficit.

In addition, results need to be discussed in terms of conditioning models of food cravings. For example, it has been suggested that repeated consumption of craved foods in the presence of hunger would increase the number of food cravings (Gibson and Desmond, 1999; Martin et al., 2006). Under this assumption, it would be expected that preventing consumption of craved foods when hungry leads to an attenuation of learned hunger-craving associations and, thus, to a decrease in food cravings. As participants maintained their usual food intake other than chocolate during the deprivation phase in the current study, they likely experienced their usual daily fluctuations in hunger levels as well. Thus, the increase in cravings as a result of preventing consumption of craved foods contradicts what would be suggested by conditioning approaches. However, a 2-week hedonic deprivation might be insufficient for an effect on conditioned responses (i.e., learned hunger–craving associations) and, thus, the current investigation does not represent a stringent test of the conditioning model.

By including implicit measures, the present study sought to limit reporting biases in explicit measures by testing whether implicit measures would show a similar pattern in the course of hedonic deprivation. Previous studies have indeed shown that such implicit preference measures are sensitive to food deprivation and hunger manipulations (Seibt et al., 2007; Stafford and Scheffler, 2008). While high trait chocolate cravers showed higher implicit chocolate preference than low trait chocolate cravers in the current study, this implicit preference for chocolate was unaffected by hedonic deprivation. One possible explanation may be that the implicit measures used in the current study capture a more trait-like chocolate preference that is relatively stable over time. Food preferences are driven by associative learning mechanisms that were likely established during repeated experiences in childhood (Ventura and Worobey, 2013). Similar to the conditioning model discussed above, a 2-week hedonic deprivation may be too short to affect implicit attitudes toward chocolate. Thus, prolonged longitudinal studies are needed to test the malleability of implicit chocolate preference measures. Another possible explanation may be that the implicit measures used in the current study might be sensitive to general need states such as hunger (Seibt et al., 2007; Stafford and Scheffler, 2008), but not to specific, hedonic processes as manipulated in the current study. This may be clarified in future studies that include both general food deprivation and specific hedonic deprivation conditions.

### Limitations

First, interpretation of results is based on a sample of young students with normal weight, which limits generalizability to individuals with higher age, lower education, under- or overweight, and clinical samples (e.g., individuals with eating disorders). Further, only a minority of participants (25% of the current sample) were male. Although chocolate craving is more common in women (Hormes et al., 2014), future studies may investigate samples with equal distribution of male and female participants to examine potential gender differences as a function of hedonic deprivation. Second, there was a relatively small number of low trait chocolate cravers included in the current study, reducing statistical power to detect between-group differences as a function of hedonic deprivation. For example, a trait × state interaction for the SC-IAT fell short of significance, which descriptively pointed toward increased positive implicit attitudes toward chocolate after deprivation in high trait chocolate cravers. Thus, whether implicit attitudes toward chocolate may indeed be influenced by chocolate deprivation needs to be further examined in future studies with larger sample sizes. Finally, while the deprivation period of 2 weeks used in the current study corresponds well with those applied in previous studies on hedonic deprivation (Moreno-Domínguez et al., 2012), a longer deprivation period may be investigated in order to determine if an increase in craving for the deprived food may be superseded by a decrease in craving after a certain amount of time.

## Conclusion

By disentangling homeostatic and hedonic effects of dieting, the present findings support the potentially causal role of hedonic deprivation in the occurrence of food cravings and underscore the special role of trait food craving. Thus, it appears that the occurrence of food cravings during dieting may be explained by (self-)deprivation rather than by an actual caloric deficit (Lowe and Levine, 2005). Results further support the rationale of existing therapeutic approaches for binge eating-related disorders and obesity that encourage flexible and moderate food consumption with no forbidden foods (Wilson et al., 2007). Indeed, rigid dieting strategies relate to more food cravings, which in turn relate to low dieting success (Meule et al., 2017; Meule, in press). This implies that rigid diet plans ought to be replaced by flexible dieting strategies that are permissive for craved foods under certain quota. Such strategies seem to be particularly appropriate for individuals with a higher susceptibility to experience food cravings (i.e., high levels of trait food craving). Therefore, food cravings need to be considered as a key factor in individuals’ difficulties to regulate or maintain a healthy diet and weight. For example, monitoring changes in trait food craving during or after diet-related interventions may be useful for predicting treatment outcomes or relapse (e.g., binge eating or weight re-gain) in patients with eating- and weight disorders.

## Author Contributions

JB and MF conceived this study. AR analyzed the data and drafted the manuscript. AM, JB, and MF contributed to interpretation of the data and revised the manuscript for content.

## Conflict of Interest Statement

The authors declare that the research was conducted in the absence of any commercial or financial relationships that could be construed as a potential conflict of interest.

## Footnotes

1 Picture numbers in the food.pics database: 0056, 0159, 0189, 0289, 0290, 0291, 0293, 0441, 0501, and 0506; Positive words: vacation [Urlaub], celebration [Feier], freedom [Freiheit], joy [Freude], peace [Frieden], gift [Geschenk], happiness [Glück], laughter [Lachen], love [Liebe], summer [Sommer]; Negative words: fear [Angst], grief [Trauer], hatred [Hass], accident [Unfall], pain [Schmerz], violence [Gewalt], enemy [Feind], evil [Böses], war [Krieg], loss [Verlust] (retrieved from https://implicit.harvard.edu/implicit/germany). German translations are provided in brackets.

2 Picture numbers in the food.pics database: 0083, 0127, 0140, 0161, 0162, 0163, 0165, 0167, 0169, 0170, 0172, 0173, 0174, 0286, and 0287 (chocolate pictures); 0407, 1038, 1039, 1043, 1041, 1042, 1044, 1045, 1046, 1047, 1048, 1049, 1050, 1051, and 1052 (object pictures).

3 The current procedure also included a recent extension of the AMP, comprising a long ISI condition (1500 ms). This version of the AMP provides not only an indicator of spontaneous affect elicited by primes, but also an indicator of spontaneous affect regulation in the long relative to the short ISI condition (Hofmann et al., 2009a, 2010). However, *ISI* (1500 ms vs. 100 ms) did not vary as a function of *trait* (high vs. low trait chocolate craving), *stimulus category* (chocolate vs. neutral primes), or *state* (deprived vs. non-deprived), all *F*_s_ ≤ 1.66, all *p*_s_ ≥ 0.203, all $\eta_{\mathrm{ps}}^{2}$ ≤ 0.031, and, thus, was not considered in the analyses (see Statistical Analyses).
